# DL-TODA: A Deep Learning Tool for Omics Data Analysis

**DOI:** 10.3390/biom13040585

**Published:** 2023-03-24

**Authors:** Cecile M. Cres, Andrew Tritt, Kristofer E. Bouchard, Ying Zhang

**Affiliations:** 1 Department of Cell and Molecular Biology, College of the Environment and Life Sciences, University of Rhode Island, Kingston, RI 02881, USA; 2 Lawrence Berkeley National Laboratory, Scientific Data Division, Berkeley, CA 94720, USA; 3 Lawrence Berkeley National Laboratory, Applied Mathematics & Computational Research Division, Berkeley, CA 94720, USA; 4 Lawrence Berkeley National Laboratory, Biological Systems & Engineering Division, Berkeley, CA 94720, USA; 5 Redwood Center for Theoretical Neuroscience, Helen Wills Neuroscience Institute, University of California, Berkeley, CA 94720, USA

**Keywords:** deep learning, DNA sequencing, read classification, metagenomics

## Abstract

Metagenomics is a technique for genome-wide profiling of microbiomes; this technique generates billions of DNA sequences called reads. Given the multiplication of metagenomic projects, computational tools are necessary to enable the efficient and accurate classification of metagenomic reads without needing to construct a reference database. The program DL-TODA presented here aims to classify metagenomic reads using a deep learning model trained on over 3000 bacterial species. A convolutional neural network architecture originally designed for computer vision was applied for the modeling of species-specific features. Using synthetic testing data simulated with 2454 genomes from 639 species, DL-TODA was shown to classify nearly 75% of the reads with high confidence. The classification accuracy of DL-TODA was over 0.98 at taxonomic ranks above the genus level, making it comparable with Kraken2 and Centrifuge, two state-of-the-art taxonomic classification tools. DL-TODA also achieved an accuracy of 0.97 at the species level, which is higher than 0.93 by Kraken2 and 0.85 by Centrifuge on the same test set. Application of DL-TODA to the human oral and cropland soil metagenomes further demonstrated its use in analyzing microbiomes from diverse environments. Compared to Centrifuge and Kraken2, DL-TODA predicted distinct relative abundance rankings and is less biased toward a single taxon.

## 1. Introduction

A microbiome defines a community of microorganisms and their activities in a given environment. This term encompasses the microbial species themselves, but also the collection of molecules they produce such as metagenomes [1,2]. Microbiome studies can be useful to different fields such as medicine or environmental protection. For example, the human gut microbiome is being extensively analyzed to uncover how its composition is linked to various disorders [3], while the ocean microbiome provides information on the potential impact of climate change on marine biodiversity [4].

The metagenomic study of microbiomes has gained a lot of interest due to the progress made in DNA sequencing technology. While the history of DNA sequencing started a decade after many proteins were already sequenced and RNA sequencing was being apprehended [5], it quickly evolved in the late 1970s when Sanger and Gilbert independently developed methods that allowed sequencing of 50 and 100 nucleotides, respectively [6,7]. The automation of Sanger’s technique combined with the desire to sequence large fragments of DNA brought various improvements that led to the development of efficient machines able to perform DNA sequencing in a particularly parallel fashion. Current high-throughput sequencing methods can produce billions of DNA fragments simultaneously during a single run. In addition, high-throughput sequencing technology offers speed and a decrease in cost per base, but also offers high sequencing depth that comes with better sensitivity. This provides the means to study uncultivable microorganisms and to detect low abundance microorganisms of a microbial community.

In a typical metagenomic study, the genetic material in all organisms contained in a given sample is fragmented, and the DNA fragments sequenced are identified as reads. Following sequencing, diverse bioinformatic tools are used to remove low-quality sequences and to assemble overlapping reads into contiguous DNA segments, also called contigs. Contigs are then arranged through scaffolding into longer segments to eventually reconstruct genomes present in the sample. This complex process of de novo sequence assembly is further challenged when dealing with short-read sequences and the high sequencing depth that is required to differentiate similar or repetitive sequences. The recent development of third-generation sequencing platforms enabled the determination of long-read sequences. With a length of 10–25 kb [8] from the Pacific Biosciences (PacBio) and 10–100 kb from Oxford Nanopore Technologies (ONT) platforms, de novo assembly will be greatly facilitated and improved as it can already be seen [9,10].

A complementary approach to analyze metagenomic data and provide information on the composition of microbial communities is the taxonomic classification of reads. This method involves assigning a taxonomic group to every read with the goal of classifying as many sequences as possible and identifying species present in the sample. One strategy for taxonomic classification consists of comparing k-mer signatures in metagenomic reads to a database of categorized k-mers. One of the state-of-the-art tools for metagenomic classification is Kraken [11], which relies on a database of k-mers with each k-mer associated with the lowest common ancestor of all genomes containing that specific k-mer. Kraken has been criticized for employing a memory-intensive algorithm [12,13,14], prompting its designers to release Kraken2, which features a more memory-efficient data structure [15]. An alternative method to efficiently store and query the database of k-mers is a modified implementation of the FM-index, employed by Centrifuge [16]. Both Kraken2 and Centrifuge have been praised in the literature for providing high accuracy and rapid runtimes [13,17].

The rapid development of deep learning techniques has inspired new applications in the analysis of metagenomic data. Deep learning models rely on artificial neural networks designed based on the structure and function of neurons in the human brain [18]. Complex deep learning models containing many layers have the ability to extract relevant features and find abstract patterns in data, allowing them to achieve high accuracy. For example, the desire to push forward the capacities of deep neural networks has led to the development of new techniques and architectures to classify images, which can reach an accuracy of 99.84% [19] on the MNIST handwritten digit classification dataset.

The first study to consider deep learning algorithms in the classification of DNA sequences built a convolutional neural network (CNN) to classify 16S small subunit ribosomal RNA (rRNA) genes which are commonly used for the identification of bacteria [20]. This study employed the bag of words technique to represent reads simulated from 16S rRNA reference sequences in terms of k-mer occurrences, thus obtaining sparse matrices as input vectors for their neural network. A k-mer size of 7 was used to restrict the storage and computational complexities that occur with sparse input vectors. Despite these limitations, they reported an accuracy of 91% across 100 bacterial genera on an artificial validation dataset. Another CNN method was proposed to classify short reads of 16S rRNA genes across 2768 genera, and achieved better sensitivity compared to Kraken2 at the genus level on 100 bp and 200 bp synthetic reads generated using 16S rRNA genes as templates [21]. While the tools mentioned above would support a high classification rate with amplicon sequencing data that targets specific genetic regions such as the 16S rRNA genes, other software have been designed to analyze the entire genetic materials sequenced from a sample. One such method called GeNet shows the improvement of training a CNN model with long DNA sequences by recording better classification of long metagenomic reads from a mock community consisting of ten microbial species, with comparable performances with Kraken and Centrifuge at the species and genus levels [22]. A more recent tool called DeepMicrobes targets 2505 bacterial species from the human gut and implements a bidirectional long short-term memory (LSTM) in addition to a self-attention mechanism [23]. DeepMicrobes outperforms other traditional taxonomic classification tools at the genus level on mock communities, suggesting the potential of LSTM in metagenomic read classification. However, LSTM is significantly slower than CNN. Finally, a recent model called BERTax, based on the state-of-the-art model BERT for natural language processing, classifies DNA sequences at the superkingdom, phylum, and genus taxonomic levels and shows generalization on unknown data compared to other approaches mentioned previously [24]. For a more in depth analysis of the deep learning techniques applied to taxonomic classification, we recommend a review published recently by [25].

Here, we present DL-TODA, a deep learning model based on CNN that classifies short metagenomic reads from over 3000 bacterial species. Compared to the aforementioned tools, DL-TODA is trained with a modified version of the deep neural network AlexNet, a successful CNN in computer vision. A training dataset containing 250 bp reads simulated from all complete bacterial genomes available in the NCBI Reference Sequence database was used for training the DL-TODA model. This enabled the identification of bacteria originating from a wide range of free-living and host-associated habitats. DL-TODA classifies each read at the species level and supports the inference of higher-order taxa based on NCBI or GTDB taxonomy. A probability score is generated for each prediction, hence permitting the quality control of prediction results based on probability thresholds.

## 2. Materials and Methods

An overview of all steps involved in the training, validation, and testing of the DL-TODA model is presented in Figure S1. Below, we provide detailed descriptions of the corresponding steps.

### 2.1. Bacterial Genome Selection

A total of 9859 complete bacterial genomes representing 3313 different species isolated from diverse free-living and host-associated environments were selected from the genome taxonomy database (GTDB) release 95 and the NCBI RefSeq database, downloaded on 7 March 2020. The genomes selected are not derived from metagenome or environmental samples and have a size equal to or above 500 kb. For each species, 70% of the genomes were randomly assigned for model training and the remaining 30% for model testing. In the cases of species with a single genome, the genome in question was automatically appointed to the training set. Additionally, all representative genomes from GTDB were automatically assigned for training. In total, we have 7405 and 2454 genomes assigned for training and testing, respectively. Table 1 provides a summary of the number of taxa represented at species, genus, family, order, class and phylum levels in training, validation and testing sets, and for both GTDB and NCBI taxonomy. A smaller number of taxa are represented in the NCBI classification due to different assignments with the GTDB classification. For example, amongst the 537 genomes classified as *Escherichia coli* by NCBI, 363, 93, 80 and 1 genomes are assigned by GTDB to *Escherichia flexneri*, *Escherichia coli*, *Escherichia dysenteriae,* and *Escherichia coli*_C, respectively. Additionally, 244 genomes lack specific assignments in at least one of the given taxonomic ranks in the NCBI taxonomy database. For example, genome GCA_000317655.1 is not assigned a class in the NCBI taxonomy but is assigned to the class of Cyanobacteria in the GTDB taxonomy.

### 2.2. Reads Simulation

Paired-end reads of 250 bp were simulated using ART Illumina read simulator (version 2.5.8) [26]. A coverage of 7 and 3 was used for read simulations using training and testing genomes, respectively. A mean fragment length of 300 bp and a standard deviation of fragment length of 10 bp were chosen according to ART Illumina usage information. The built-in error profile of MiSeq v1 (MSv1) was used for simulation. The command for running ART Illumina is art_illumina -ss ‘MSv1’ -i <input fasta file> -d <reads prefix id> -na -s <standard deviation of fragment length> -m <mean fragment length> -l <read length> -f <fold coverage> -p -o <output file>.

### 2.3. Training, Validation and Testing Sets

**Figure 1:**
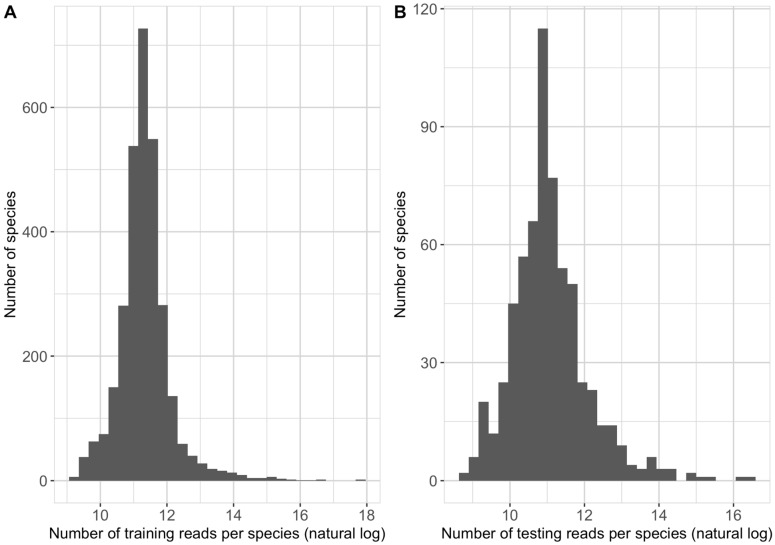
Distribution of number of training (**A**) and testing (**B**) reads per species based on the NCBI taxonomy in the natural log scale.

Paired-end reads obtained from training genomes were randomly shuffled and split into 70% for training and 30% for validation. The forward and reverse reads from testing genomes were treated separately and classified independently. Identical reads between the training and testing data were identified by clustering the training and testing reads using Mmseqs2 easy-linclust (version 13.45111) with a minimum sequence identity of 1.0 and a fraction of aligned residues of 1.0. To avoid biases in testing, testing reads that are identical to the training reads were removed from the testing set. Table 2 summarizes the final number of reads included in the training, validation and testing sets of this study. The number of training reads allocated to each species in the NCBI taxonomy had a median of 80,067 and ranges between 10,359 and 56,838,380 (Figure 1A). The number of testing reads allocated to each species in the NCBI taxonomy had a median of 56,839 and ranges between 6455 and 14,223,296 (Figure 1B). The genome coverage represented in the training data was calculated for each species based on Equation (1), where the “number of training reads” are the number of reads assigned to a given species label in the training data, and the “average training genomes size” accounts for the average length of training genomes of the given species.

(1) genome coverage=250 ∗number of training reads / average training genome size

### 2.4. Deep Learning Neural Network

#### 2.4.1. Reads Representation

DL-TODA represents each read as a vector of k-mers, using a sliding-window of size 12 across the 250 bp read sequence. Reads shorter than 250 bp were padded with 0s before representation of the k-mers. A vector of 239 integers was then used to represent each read based on a k-mer size of 12 and an indexed vocabulary of 12-mers (described in the section below). The read vectors were then stored in TensorflowRecord (TFRecord) files alongside labels corresponding to the species assignment (i.e., ground truth), and presented to the embedding layer.

#### 2.4.2. K-mer Embedding

The DL-TODA model embeds each k-mer by choosing only the canonical form in a pair consisting of the k-mer and its reverse complement. The canonical k-mer corresponds to the k-mer that appears first, according to the alphabetical order. This strategy allows us to reduce the vocabulary learned by the neural network and therefore lower the complexity of the model. The number of all possible canonical 12-mers is 8,390,656, defined as $\frac{\left(4^{k}+4^{\left(k/2\right)}\right)}{2}\;\left(k=12\right)$. The vocabulary of DL-TODA included all the canonical 12-mers and two additional digits, one accounting for unknown 12-mer with characters different from the four universal bases (i.e., A, T, G, C), and another for padded 0s to the right of sequences shorter than 250 bp. Following the vocabulary definition, each 12-mer was assigned an index between 0 and 8,390,657 in order to retrieve a vector of 60 real values from a list. These vectors were initiated in the Tensorflow embedding layer, with each real value drawn from the He Normal distribution [27], and were updated during training.

#### 2.4.3. DL-TODA Neural Network

**Figure 2:**
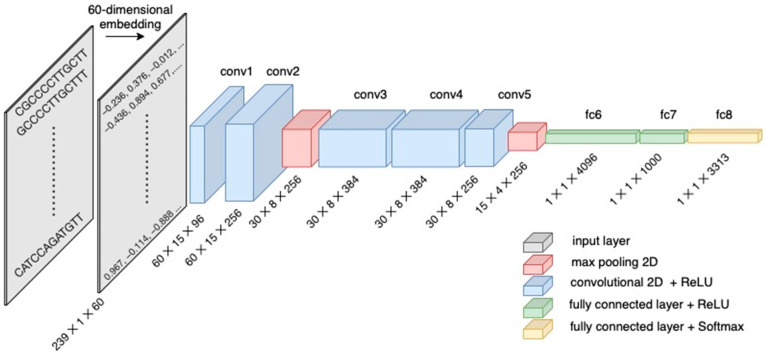
Convolutional neural network architecture used to build the taxonomic read classifier DL-TODA. Each read is represented as an input layer (239 × 1 × 60) by embedding 12-mers into vectors of 60 real values. The input layer is then processed by five convolutional layers, two max pooling layers and three fully connected layers.

The deep neural network architecture of DL-TODA is a modified version of AlexNet [28] (Figure 2) with a trainable embedding layer generating an (8,390,658 × 60) embedding matrix. The input layer of this neural network is a (239 × 60) matrix consisting of 239 rows of 12-mers embedded as 60 real value vectors (described above). The input data are then processed by five convolutional layers, two max pooling layers and three fully connected layers. The rectified linear unit (ReLU) activation function is applied throughout the neural network, except in the last layer, in which the softmax function transforms the output from the fully connected layer to a probability distribution over the 3313 species.

#### 2.4.4. Loss Function and Probability Scores

The following cross entropy loss function (Equation (2)) was used to compute the difference between the species desired output (0 or 1) and the estimated probability of correct prediction for a given species for one example.

(2) $$Cross\;Entropy\;Loss=-\overset{3313}{\underset{i=1}{\sum }}actual\;value\;of\;Species_{i}*log\left(predicted\;probability\;of\;Species_{i}\right)$$

The estimated probability of every species is obtained by applying the softmax function [29] to an output vector of 3313 real numbers.

### 2.5. Training and Testing

**Figure 3:**
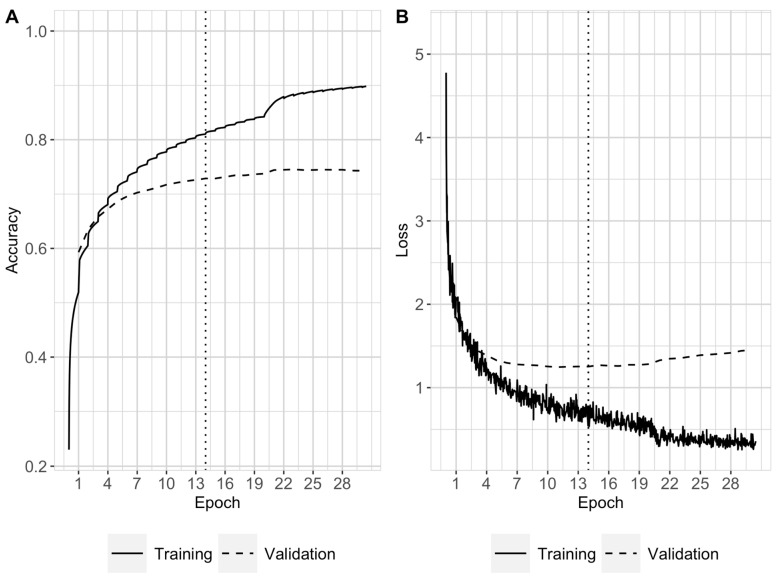
Learning curves representing the predictive performance of DL-TODA during training in terms of accuracy (**A**) and loss (**B**). The training loss and accuracy (solid line), validation loss and accuracy (dashed line), and epoch 14 at which the model was tested (dotted line) are reported.

Data loading to the neural network was performed using the Nvidia Data Loading Library (DALI). Shuffling was carried out exclusively for the training and validation sets. Distributed training was executed by dispatching batches of 512 reads to four different GPUs (global batch size of 2048). Each GPU computed gradient updates independently; these were then averaged together and finally applied to the model. The accuracy and loss computed with the training and validation sets were monitored and saved throughout the training to create learning curves (Figure 3). Additionally, the model was saved at the end of every epoch. Testing and applications to the oral and soil metagenomes were carried out similarly with a batch size per GPU of 512 reads distributed among four GPUs and using the trained model saved at epoch 14.

### 2.6. Evaluation of Model Performance

The performance of DL-TODA was assessed with the overall classification accuracy, defined in Equation (3), at different taxonomic ranks including species, genus, family, order, class and phylum.

(3) Accuracy=# reads correctly classified / # reads classified

At the species level, the number of correctly classified reads was directly obtained from the neural network. At higher taxonomic ranks, the number of correctly classified reads was calculated with the sum of all reads that were correctly assigned to the species within each taxon.

**Figure 4:**
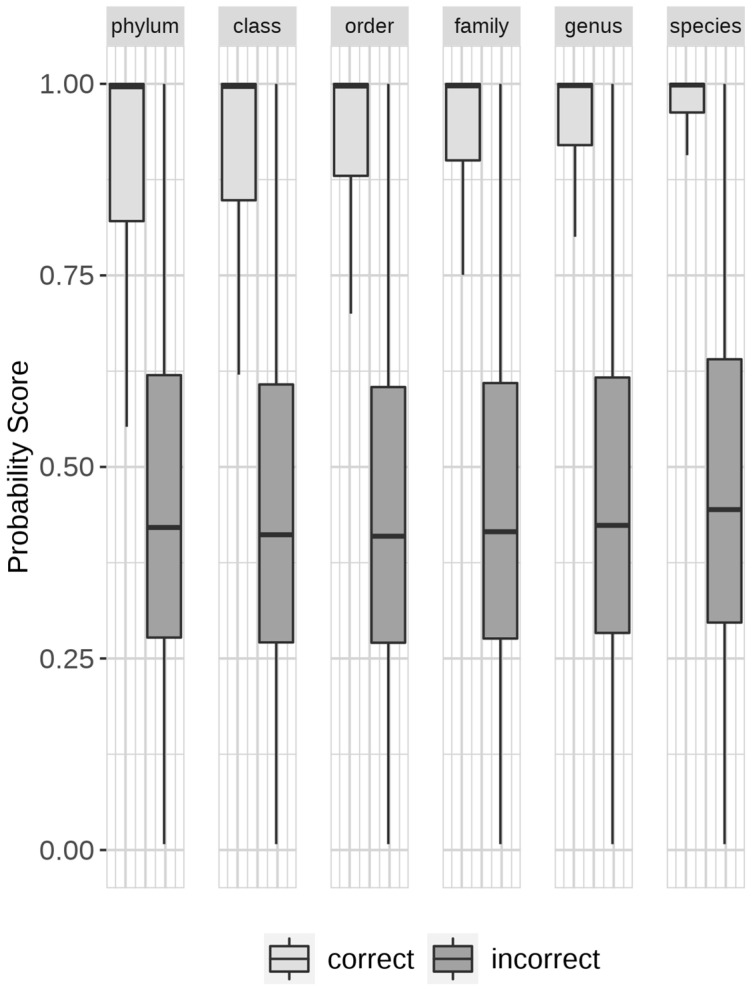
Distribution of probability scores in DL-TODA for correct and incorrect predictions obtained on the entire testing set. The visualization is made in the form of a box plot. The median values are indicated with a thick horizontal line in the rectangle boxes. Lower and upper edges of the rectangle boxes indicate the first and third quartiles, respectively. The thin vertical lines indicate the upper and lower whisker limits, defined as Q3+1.5×IQR and Q1−1.5×IQR, respectively, where Q1 is the first quartile, Q3 is the third quartile, and IQR is the inter quartile range from Q1 to Q3. Outlier data points beyond the upper and lower whisker limits are not shown in the box plot.

The percentage of classified vs. unclassified reads was also examined with the application of different thresholds on the predicted probability of species. The selection of threshold settings was guided by the overall distribution of probability scores among the correct or incorrect classification in the testing dataset (Figure 4). The eqgamma function of the R package EnvStats (version 2.7.0) was used for identifying confidence intervals based on a gamma distribution for the elimination of incorrect predictions. The precision (Equation (4)), recall (Equation (5)) and F1-score (Equation (6)) were obtained for each species. The macro and micro average of each metric (Equations (7)–(12)) were computed to provide a comparison of the performance between DL-TODA, Kraken2 and Centrifuge. The number of true positives (TP), false positives (FP) and false negatives (FN) per species required to compute precision, recall and F1-score were obtained based on the generation of a confusion matrix.

(4) Precision=TP/(TP+FP)

(5) Recall=TP/(TP+FN)

(6) F1−score=2∗ Precision ∗ Recall/(Precision+Recall)

(7) Macro average precision =sum of Precision for each species/number of species

(8) Micro average precision=sum of TP/(sum of  TP+ sum of FP)

(9) Macro average recall=sum of Recall for each species/number of species

(10) Micro average recall=sum of TP/(sum of  TP+sum of FN)

(11) Macro average F1−score=sum of F1−score for each species/number of species

(12) Micro average F1−score=sum of TP/(sum of  TP+1/2 ∗(sum of FN+sum of FP))

### 2.7. Comparison with Kraken2 and Centrifuge

We evaluated the performance of DL-TODA in comparison with Kraken2 version 2.0.8 and Centrifuge version 1.0.3. For both programs, an index was built with the training genomes as references to classify the simulated reads in the testing set using the default settings. Given that both Kraken2 and Centrifuge classify reads to the NCBI taxonomy database, we used the NCBI taxonomy for analyzing the results from DL-TODA. Centrifuge provides multiple possible predictions per pair of reads or unpaired reads. Here, the top hit was systematically used as the predicted taxon.

### 2.8. Classification of Metagenomic Data

The functionality of DL-TODA was determined by classifying metagenomes obtained from sampling two distinct environments, human oral cavity and cropland soil. The human oral cavity datasets were identified following [30]. The cropland soil datasets (NCBI accessions: ERR5004682, ERR5003895, ERR5003204, ERR5001925 and ERR4995171) were identified from the National Microbiome Data Collaborative (NMCD) data portal [31], using “soil” as the keyword for ecosystem type and “cropland ecosystem” as the keywords for broad-scale environmental context. The metagenomic reads were retrieved using the SRA Toolkit from NCBI, converted to TFRecords and classified by DL-TODA with a probability score threshold above 0.5 (i.e., reads with probability scores below or equal to 0.5 were counted as unclassified). The relative abundance of each taxon was measured by dividing the number of reads classified to that taxon by the total number of reads in the metagenome (Equation (13)). The DL-TODA classification was compared with Kraken2 and Centrifuge classifications of the same metagenomes, using the training genomes as references.

(13) Relative Abundance=number of reads classified to a taxon / total number of reads

### 2.9. Computational Requirements

The DL-TODA model was trained and tested on a compute node with 768 GB of High Performance DDR4 2666 MHz ECC system memory, 48 Intel Xeon Cascade Lake Scalable Cloud Ready Processor Cores/2.2 GHz processors and four Nvidia A100/40 GB HBM2 Memory GPUs. Kraken2 and Centrifuge were run on a compute node with 24 Intel(R) Xeon(R) CPU E5-4607 0/2.20 GHz processors and 512 GB of memory. The deep learning model was implemented with TensorFlow as a Python3 script, Horovod was used to distribute training across multiple GPUs and the Nvidia DALI was used to load the TFRecord files.

## 3. Results

### 3.1. Model Training and Testing

Training of DL-TODA was conducted on a GPU node with four GPUs and was terminated when the model had reached 31 epochs, as no improvements in the validation accuracy were observed (Figure 3A). The model saved at epoch 14 was subsequently selected to perform testing on the testing set, as the model started memorizing the training data after that point, as shown by the progressive increase in the validation loss (dashed line on Figure 3B). Furthermore, additional testing carried out at other checkpoints did not show significant accuracy improvement.

DL-TODA is designed to provide a vector of probability scores in the prediction of every read, with each score corresponding to the probability that the read should be assigned to a given taxon. A taxon with a score of 0.5 has an equal probability of being the true or false assignment of the read analyzed, while a score between 0.5 and 1.0 gives a higher confidence that the read can be truly assigned to the taxon. The DL-TODA prediction of each testing read was designated as either correct or incorrect based on whether the highest probability score was assigned to the ground truth taxon. Of the 109,851,839 reads tested, over 82%, 88%, 90%, 92%, 94%, and 96% were correctly assigned to the corresponding ground truth taxa at the taxonomic ranks of species, genus, family, order, class, and phylum, respectively. The distributions of probability scores among correct and incorrect classifications were plotted in Figure 4. The probability scores of incorrect predictions had median values under 0.5 across all taxonomic ranks, aligning with the expectation that a probability of 0.5 or lower represents predictions with low confidence. In contrast, the probability scores of correct predictions had median values above 0.99 for all taxonomic ranks, and the 25th percentile ranging from 0.82 at the phylum level to 0.96 at the species level. Given the high number of correct taxonomy assignments even with the simple application of top-ranking probability scores, along with the observed clear separation of probability score distributions among correct predictions compared to incorrect predictions, we hypothesize that a decision threshold can be applied on the top-ranking probability scores to further enhance the prediction accuracy of DL-TODA.

### 3.2. Optimization of Probability Threshold

**Figure 5:**
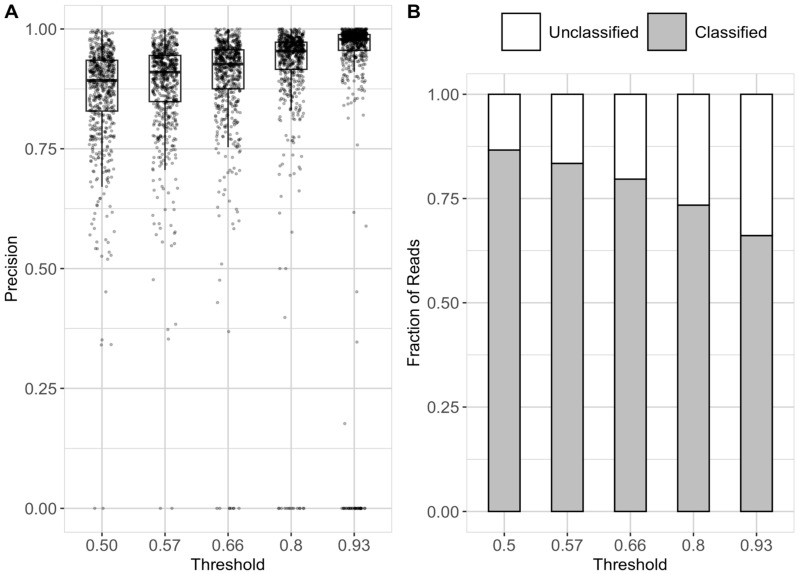
DL-TODA precision for the 639 species in the testing set (**A**) and fraction of unclassified and classified reads at the species level (**B**) at different decision thresholds (0.5, 0.57, 0.66, 0.8 and 0.93).

To guide the selection of an optimal threshold, we visualized the species-level precision of DL-TODA predictions in the testing data, given a series of cutoff values. The probability scores below 0.5, 0.57, 0.66, 0.8 and 0.93 correspond to 60%, 70%, 80%, 90% and 95% of incorrect predictions, respectively, based on fitting a gamma distribution over the probability scores of the incorrect assignments. The elimination of low confidence assignments (by assigning predictions only to reads with probability score higher than a designated threshold) greatly enhances the overall precision of DL-TODA predictions for the 639 species tested (Figure 5A). With a threshold of 0.93, the median precision across all species was 0.98, which is 9% higher than the median precision of 0.89 obtained with a threshold of 0.5. The higher thresholds, however, could potentially limit the number of classified reads. Of the thresholds tested, the percentage of classified reads ranged from 87% under 0.5 to 66% under 0.93 (Figure 5B). To balance the gains of precision on species-level predictions and the losses on the number of classified reads, we decided to choose a threshold of 0.8, which gives a median precision of 0.95 across the individual species while still classifying 73% of all the testing reads with high confidence.

Despite the overall high performance, DL-TODA obtained relatively low precision scores in the prediction of a small number of species (Figure 5A). A close examination of these poorly predicted species revealed that each species was represented by only one or a few genomes in the training data, suggesting a general lack of training depth in the deep learning model. Figure S2 elucidates the correlations between training genome coverage and model performance. With genome coverage higher than 55 ($\sim e^{4}$), DL-TODA consistently reported high precision (e.g., greater than 0.75) in the prediction of corresponding species. Under lower training coverage, however, the minimum precision scores were positively correlated with the training coverage. It was also noted that many species, despite having a training genome coverage of less than 7 ($\sim e^{2}$), achieved high precision of above 0.9, suggesting that a high coverage is not required for all species in the DL-TODA training.

### 3.3. Comparison with Kraken2 and Centrifuge

**Figure 6:**
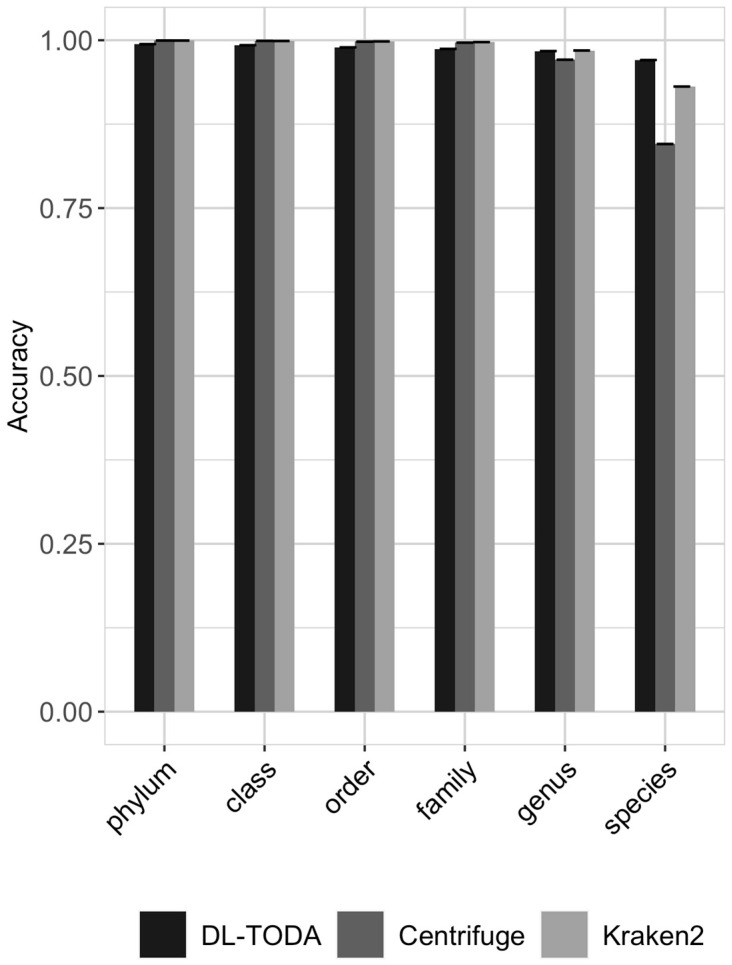
Accuracy across taxa at different taxonomic ranks obtained by running DL-TODA, Centrifuge and Kraken2 on ten subsets of the testing set. The error bar is plotted at the top of each bar. Results for DL-TODA are reported in the presence of a decision threshold of 0.8.

Kraken2 and Centrifuge were applied to the same testing set to assess the performance of DL-TODA amongst taxonomic classification tools. Both Kraken2 and Centrifuge require the construction of reference databases. In order to make a fair comparison, all genomes seen by DL-TODA during training were used to build the indexed reference database for both tools. The average accuracy obtained on ten subsets of the testing data is shown in Figure 6. The ten subsets were obtained by randomly shuffling the testing reads and splitting the testing dataset into nine subsets with 11,000,000 reads and 1 subset with 10,851,839 reads. Comparable performances were observed among all three tools at taxonomic ranks above the genus level, with the overall accuracy averaging above 0.98. At the species level, DL-TODA reached a higher average accuracy of 0.97, compared to 0.93 and 0.85, respectively, achieved with Kraken2 and Centrifuge (Figure 6). The micro average and macro average of precision, recall and F1-score obtained for the 639 species on the entire testing set are shown in Table 3. DL-TODA has higher micro average precision, recall and F1-score, which suggests that DL-TODA makes better overall predictions than Kraken2 and Centrifuge, regardless of the species compared. On the other hand, the macro average metrics for DL-TODA are lower than the corresponding micro average metrics, indicating that DL-TODA performs better for some species compared to others, especially with regard to the performance of recall. For example, with a probability threshold of 0.8, 14 species obtained a recall of 0 due to the removal of predictions with low probability scores, although the majority of other species were predicted with high precisions (greater than 0.95) and recalls (greater than 0.85) by DL-TODA. As a contrast, Kraken2 and Centrifuge appear to manifest similar performances for all species, as their macro average metrics are largely consistent with the corresponding micro average metrics, with the exception that Centrifuge shows variability across species in terms of the recall.

### 3.4. Taxonomic Profiling of Metagenomic Data

The performance of DL-TODA on metagenomic data was assessed based on a probability threshold of greater than 0.5, using two sets of metagenomes. The first dataset was taken from the human oral microbiome [30] and the second dataset was taken from the soil microbiome [32], with a total count of 3,417,111,096 and 52,290,557 reads, respectively, for the two environments. The relative abundance of reads classified by DL-TODA, Kraken2, and Centrifuge are summarized at the species and genus levels (Table 4). In the oral microbiome, a similar percentage of metagenomic reads (20–30%) was classified by all three tools. While a similar number of taxa was identified by the three tools, DL-TODA identified the highest number of species (452 species) with a relative abundance above 0.01% over the entire set of metagenomes. This is in contrast to Centrifuge, which classified the highest percentage of reads (33%, largely driven by the assignment of classifications to read pairs) but identified a lower number of species (114 species) with a relative abundance above 0.01%. Kraken2 assigned a highest percentage of reads to unknown species compared to the other tools, suggesting a relatively low resolution at the species level. In the soil microbiome, the percentage of metagenomic reads classified by the three tools differed greatly, ranging from 20% of total metagenomic reads identified by Centrifuge to merely 4–5% identified by Kraken2. The latter also had the highest percentage of reads assigned unknown at both species and genus levels; this is similar to what was observed in the analysis of oral microbiome data. DL-TODA classified around 15% of the reads in the soil metagenome and identified 283 species with a relative abundance above 0.01%, which is slightly lower than the Centrifuge predictions but higher than the Kraken2 predictions.

**Figure 7:**
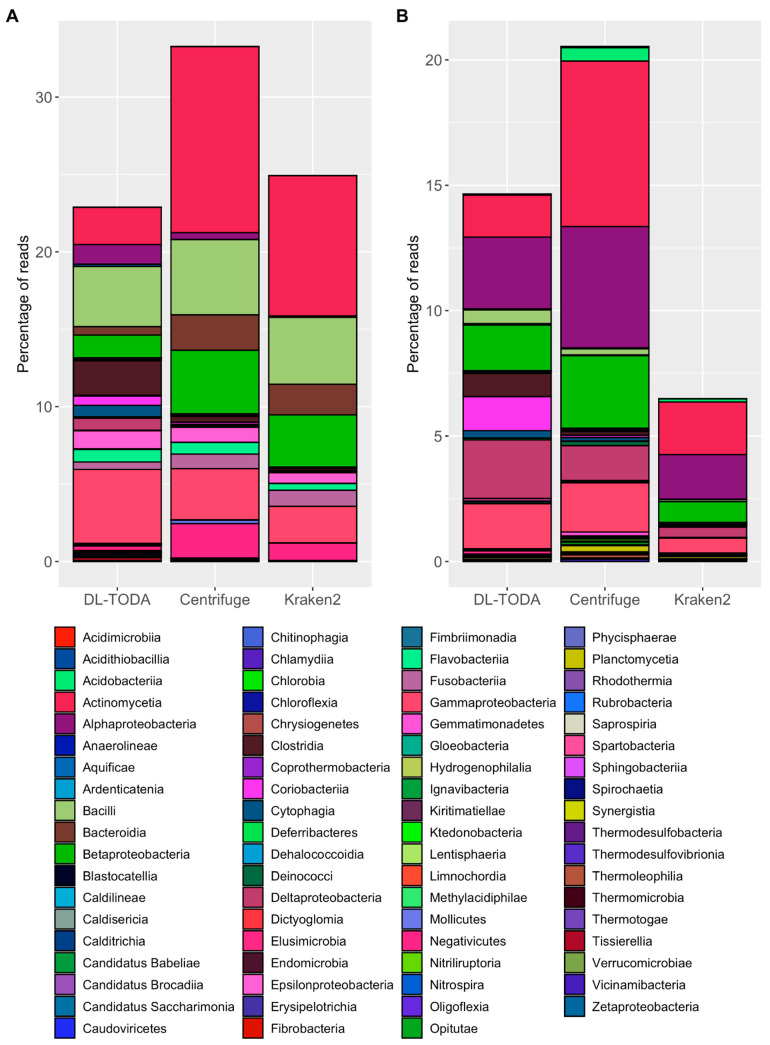
Taxonomic distribution of metagenomic reads at the class rank based on predictions made by DL-TODA, Centrifuge and Kraken2 in the human oral (**A**) and soil (**B**) metagenomes. The Y-axis indicates the percentage of reads over the entire metagenome. The two panels are color coded with the same color pallet so that the same color indicates identical taxa across the different stacked bars.

Further examination of the classification results was based on the visualization of taxonomic compositions at the class rank (Figure 7). A general consistency was observed in the predicted classes by all three tools in both the oral and soil metagenomes, while the ranking of each class’s relative abundance may vary among the different tools. The most abundant classes identified by DL-TODA in the human oral microbiome (Figure 7A) included Gammaproteobacteria (4.8%), Bacilli (3.9%), Actinomycetia (2.4%) and Clostridia (2.2%). In comparison, Clostridia was only found in a small percentage of reads (0.4% and 0.14%, respectively) by Centrifuge and Kraken2. The taxa most seen by both Centrifuge and Kraken2 are Actinomycetia (12.0% and 9.1%), Bacilli (4.9% and 4.3%), Betaproteobacteria (4.1% and 3.4%), Gammaproteobacteria (3.3% and 2.4%) and Bacteroidia (2.3% and 2%). The results obtained with the soil metagenome show similar trends. Kraken2 and Centrifuge manifest similar outcomes with Kraken2 classifying a much lower number of reads (Figure 7B). Actinomycetia, Alphaproteobacteria, Betaproteobacteria and Gammaproteobacteria are amongst the top-ranking classes observed by Kraken2 and Centrifuge, with relative abundances ranging from 0.6% to 6.6%. These bacterial taxa are also predicted by DL-TODA with different relative abundances varying between 1.7% and 2.9%. Additionally, DL-TODA identified Coriobacteriia and Clostridia with relative abundances of 1.4% and 0.9%, respectively, while the relative abundance for Coriobacteriia was 0.08% with Centrifuge and 0.02% with Kraken2, and the relative abundance for Clostridia was 0.15% with Centrifuge and 0.05% with Kraken2.

## 4. Discussion

Taxonomic classification of billions of short sequencing reads is an important step in the analysis of metagenomic data, shedding light into the function and diversity of microbiomes. Such analysis can be performed by several existing programs but still has room for improvement. K-mer based approaches, such as Kraken2 and Centrifuge, are the most common strategies to classify metagenomic data. While both Kraken2 and Centrifuge rely on the construction of reference databases, the use of a deep learning model in DL-TODA permits the extraction of features during model construction, hence circumventing the requirement of a reference database.

An accuracy similar to higher classification was achieved by DL-TODA compared to Kraken2 and Centrifuge on an independent test set of over a hundred million simulated metagenomic reads (Figure 6). A look at the precision, recall and F1-score (Table 3) further demonstrated the better performance of DL-TODA, as it carried a higher micro average on all three metrics compared to Kraken2 and Centrifuge. However, lower macro than micro averages were observed in DL-TODA, indicating potential differences in how well it recognizes different species. In contrast, Kraken2 and Centrifuge appeared to perform more equally across species, as their macro average metrics are comparable to the corresponding micro average metrics.

One possible reason why DL-TODA may have performed poorly on some species may be the lack of sufficient training data. This is supported by the positive correlations between depth of training genome coverage and minimum precisions observed (Figure S2). For example, when the coverage is greater than 55 ($\sim e^{4}$), the precision values are consistently higher than 0.75, suggesting that a higher and potentially more diverse set of training data may lead to an enhanced performance of DL-TODA. However, we note that some species, despite having a low number of training reads, reached high precisions in DL-TODA predictions. This may indicate that the DL-TODA model is efficient at extracting traits from these species for label classification. While reaching high performances on a majority of the species tested (Figure 5), DL-TODA seems to assign low probability scores to reads from a few species, resulting in low precisions approaching zero for the prediction of these species, especially when a probability threshold is used. Given the variability in the classification of different species, the probability threshold may be individually adjusted for each species to optimize the performance of DL-TODA. A careful selection of the probability threshold may require more benchmarking efforts to maximize the prediction accuracy while minimizing the fraction of unclassified data; this may be a topic of future research using diverse test cases. Future studies that seek to reveal the correlations between different genomic features (e.g., GC content, tetranucleotide frequency, distribution of mobile genetic elements, etc.) and the outcomes of read classifications can also help guide the further advancement of DL-TODA models and enhance their precision across all species.

The application of DL-TODA to the human oral and cropland soil metagenomes supports a general consensus on the prediction of top-ranking taxa, but distinct predictions on the relative abundance of different taxonomic groups compared to Kraken2 and Centrifuge (Figure 7). In the human oral metagenomes, DL-TODA identified a higher proportion of Clostridia, which is known to be abundant and diverse in the human oral microbiome [33] compared to Centrifuge and Kraken2. Likewise, in the cropland soil metagenomes, a higher proportion of Clostridia and Coriobacteriia was identified by DL-TODA compared to Centrifuge or Kraken2. The abundance of Clostridia and Coriobacteriia, as predicted by DL-TODA, aligns well with prior studies of diverse agricultural related soil types [34,35,36]. Due to the lack of ground truth data, it is difficult to fully assess the accuracy of different tools on the metagenomes. However, the Centrifuge and Kraken2 predictions seem to be highly skewed towards assigning large proportions to a small number of taxa. For example, the class Actinomycetia was assigned the highest proportions by both Centrifuge and Kraken2 in both the oral and soil metagenomes, suggesting the potential biases of Centrifuge and Kraken2 towards classifying certain taxa. In contrast, the prediction of DL-TODA is less biased towards a single taxon, and it predicted different rankings of the dominant taxa between the human oral cavity and cropland soil, two highly distinct environments. The total number of reads classified remains low across all three tools, and the percentage of classified reads varies among the two environments tested (Table 4). Large differences were observed with Kraken2, which classified over 20% of reads in the oral metagenome but only around 5% of reads in the soil metagenome. Centrifuge seems to have classified the highest proportion of reads among all three tools in both the oral and soil metagenomes. Considering that Centrifuge assigns the same taxa to paired reads, similar strategies may be employed by DL-TODA to leverage the read pairs for enhancing the number of classified reads. It is noted that the DL-TODA predictions were based on a probability threshold higher than 0.5 which was uniformly applied to all taxa. Based on discussions in the above paragraph, further optimization of the probability threshold, together with the introduction of more training data, especially for some underrepresented species, will likely further enhance the number of classified reads in the metagenomes.

Overall, DL-TODA is a new deep learning-based model for the taxonomic classification of metagenomic reads. The model showed a high accuracy in classifying synthetic reads and demonstrated the potential of recognizing a wide range of taxonomic groups from diverse environments. Besides DL-TODA, several other deep learning models have recently been created for the classification of metagenomic data, showing varied accuracy and generalizability, usually at the genus or higher taxonomic levels [23,24]. DL-TODA is distinct from these deep learning-based read classification tools. It uses a convolutional neural network designed based on the architecture of AlexNet and classifies metagenomic reads at the species level. DL-TODA has the ability to classify over 3000 bacterial species, covering all the phyla represented in the current GTDB and NCBI databases. An additional advantage of DL-TODA is the possibility to resume training with new data without needing to reanalyze the previous training sets. This allows the model to be efficiently updated with newly discovered genomes. DL-TODA also supports the calibration of classification results based on a probability score associated with each taxonomic assignment. The implementation of DL-TODA is designed to support high efficiency in processing high volumes of metagenomic data. By making use of Horovod, DL-TODA distributes the training and testing tasks across multiple GPUs in parallel, faster than with the data distribution strategy provided by TensorFlow. This feature, in addition to loading data directly to the GPU memory using the Nvidia DALI library, creates an efficient pipeline for dealing with large datasets. Future developments will include investigating solutions to reduce the size and number of parameters in DL-TODA to further accelerate the training and testing processes. Given the rapid growth of deep learning applications in metagenomic data analysis, future benchmarking studies would provide useful guidelines for the application of different deep learning tools and will likely nurture the engagement of a broader scientific community.

## Figures and Tables

**Table 1:** Taxonomic distribution of training/validation and testing datasets based on the GTDB or NCBI taxonomy databases.

	Training and Validation Sets		Testing Sets	
Database	GTDB	NCBI	GTDB	NCBI
Species	3313	3053	709	639
Genus	1414	1136	331	289
Family	465	387	138	146
Order	224	171	79	77
Class	100	74	38	33
Phylum	45	43	24	19

**Table 2:** The total number of simulated reads in training, validation and testing datasets.

Dataset	Number of Reads
Training	563,434,720
Validation	241,467,730
Testing	109,851,839

**Table 3:** Micro average and macro average of precision, recall and F1-score obtained for the 639 species in the testing set for DL-TODA, Kraken2 and Centrifuge. The DL-TODA metrics were calculated with testing reads classified with a probability score higher than 0.8.

	DL-TODA	Kraken2	Centrifuge
Micro average			
Precision	0.98	0.97	0.97
Recall	0.97	0.93	0.85
F1-score	0.98	0.95	0.90
Macro average			
Precision	0.91	0.96	0.97
Recall	0.76	0.92	0.80
F1-score	0.80	0.93	0.82

**Table 4:** Summary of species and genus level classifications made by DL-TODA, Centrifuge and Kraken2 on the human oral and soil metagenomes. The number (#) of taxa observed with relative abundances (r.a.%) ≥ 0.01% or <0.01% is reported in the table. Relative abundances represent the percentage of classified reads over the total number of reads in the metagenomes. Unknown taxa represent groups at a given taxonomic level that are not named in the NCBI taxonomy.

		Tool	# Taxa ≥ 0.01%	Sum of r.a.% ≥ 0.01%	# Taxa with r.a. < 0.01%	Sum of r.a.% < 0.01%	Sum r.a. % of Classified Reads	Unknown Taxa r.a%
oral	Species	DL-TODA	452	19.60	2571	3.76	23.35	0.0024
		Kraken2	85	20.97	2942	1.32	22.29	2.89
		Centrifuge	114	29.19	3066	4.32	33.50	0.036
	Genus	DL-TODA	281	21.27	853	1.57	22.84	0.527
		Kraken2	47	23.56	1075	0.85	24.41	0.78
		Centrifuge	111	31.22	1025	2.07	33.29	0.25
soil	Species	DL-TODA	283	11.80	2648	3.04	14.84	0.012
		Kraken2	62	1.13	2941	3.78	4.92	2.60
		Centrifuge	697	15.81	2451	4.81	20.63	0.096
	Genus	DL-TODA	206	13.11	918	1.57	14.68	0.18
		Kraken2	119	3.84	1002	1.66	5.50	2.02
		Centrifuge	345	18.65	786	1.81	20.46	0.26

## Data Availability

Source code of DL-TODA and the data presented in this study have been deposited in https://github.com/zhanglab/dl-toda (accessed on 28 February 2023) and 10.6084/m9.figshare.22184821 (accessed on 24 March 2023).

## Supplementary Materials

The following supporting information can be downloaded at: https://www.mdpi.com/article/10.3390/biom13040585/s1, Figure S1: A detailed illustration of the DL-TODA pipeline; Figure S2: Precision of DL-TODA predictions over 639 species in the testing set plotted against the depth of training set coverage for each corresponding species.
