# Supplementary material for: DL-TODA: A Deep Learning Tool for Omics Data Analysis
Source: Biomolecules. 2023 Mar 24;13(4):585. doi: 10.3390/biom13040585 (PMC10135919; doi:10.3390/biom13040585)
Supplement: Supplementary file 1 [file biomolecules-13-00585-s001.zip › biomolecules-2118772-supplementary.pdf]

SUPPLEMENTARY FIGURES:

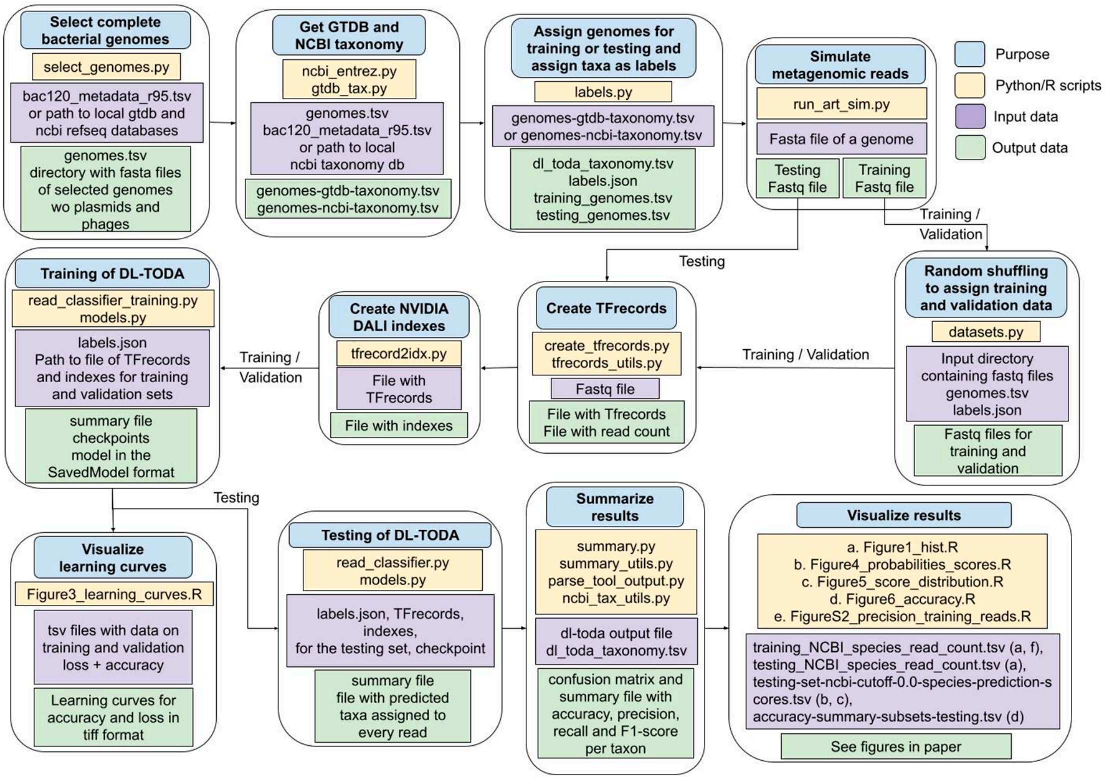

Figure S1. A detailed illustration of the DL-TODA pipeline. All complete bacterial genomes were selected from local NCBI and GTDB databases. A fasta file was created for each genome that contains chromosomal sequences, excluding plasmids or phages. In the next steps, the NCBI and GTDB taxonomies are retrieved for each selected genome followed by the assignment of each genome for training or testing purposes. Reads are then simulated for each genome with the ART Illumina read simulator and reads from genomes assigned for training are shuffled and split between training (70%) and validation sets (30%) and stored in fastq files that are then converted into TFRecords. Fastq files of simulated reads obtained from genomes intended for testing are directly converted into TFRecords. Nvidia DALI indexes are then created for each file with TFRecords. Files with training and validation TFRecords and Nvidia DALI indexes are used as input for training DL-TODA along with other accessory files. Testing is done with testing TFRecords and their corresponding Nvidia DALI indexes as well as a Tensorflow checkpoint obtained during training. R scripts were generated to visualize the distribution of training and testing reads, the learning curves, the distribution of probability scores in DL-TODA for correct and incorrect predictions, the analysis of precision for the 639 species in the testing set in DL-TODA at different decision thresholds and the accuracy at different taxonomic ranks.

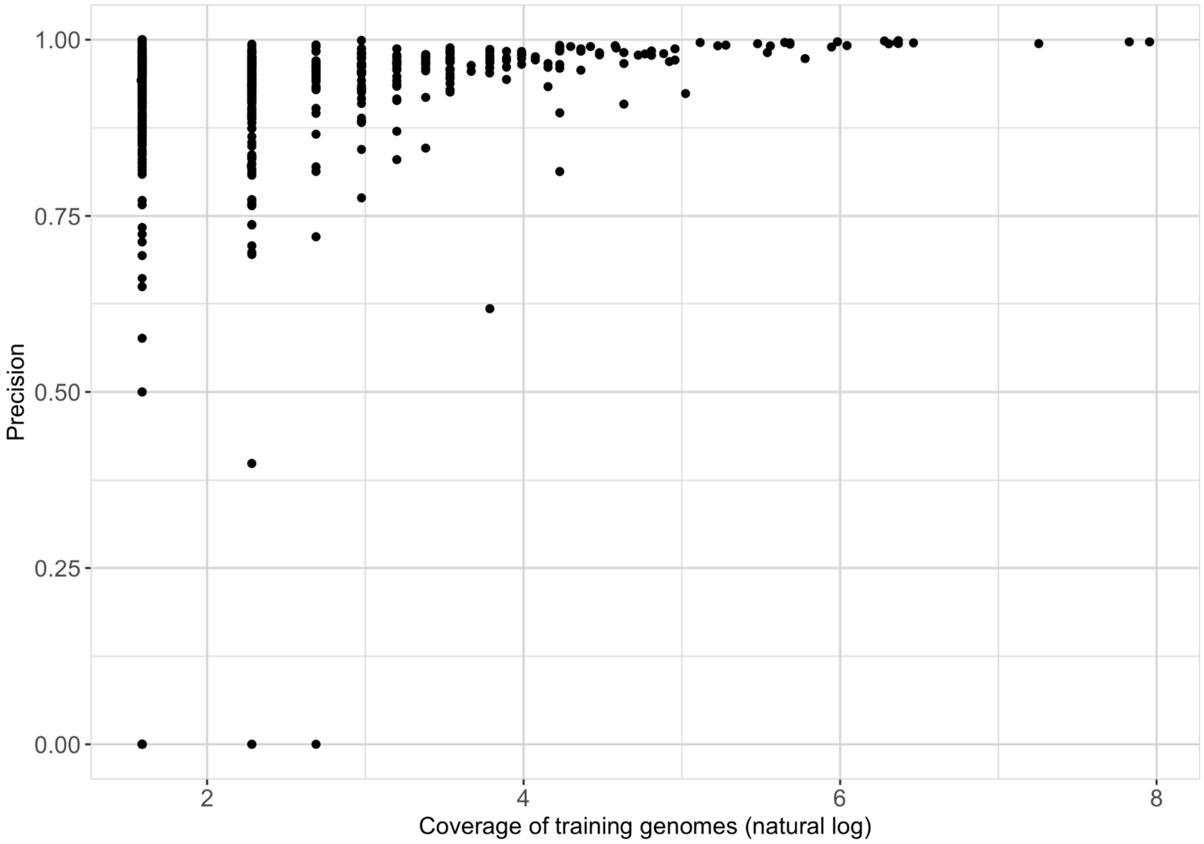

Figure S2. Precision of DL-TODA predictions over 639 species in the testing set plotted against the depth of training set coverage for each corresponding species. The precision is calculated by considering reads classified with a probability score above 0.8.
